# Preprints in times of COVID19: the time is ripe for agreeing on terminology and good practices

**DOI:** 10.1186/s12910-021-00667-7

**Published:** 2021-07-28

**Authors:** Raffaella Ravinetto, Céline Caillet, Muhammad H. Zaman, Jerome Amir Singh, Philippe J. Guerin, Aasim Ahmad, Carlos E. Durán, Amar Jesani, Ana Palmero, Laura Merson, Peter W. Horby, E. Bottieau, Tammy Hoffmann, Paul N. Newton

**Affiliations:** 1 Institute of Tropical Medicine, Nationalestraat 155, 2000 Antwerp, Belgium; 2 Infectious Diseases Data Observatory (IDDO), University of Oxford, Oxford, UK; 3 Centre for Tropical Medicine and Global Health, Nuffield Department of Medicine, University of Oxford, Oxford, UK; 4 Boston University, Boston, MA USA; 5 Howard College School of Law, University of Kwazulu-Natal, Durban, South Africa; 6 Bioethics Group, Aga Khan University, Karachi, Pakistan; 7 The Kidney Centre Post Graduate Training Institute, Karachi, Pakistan; 8 Julius Center for Health Sciences and Primary Care, University Medical Center Utrecht, Utrecht, The Netherlands; 9 Indian Journal of Medical Ethics, Mumbai, India; 10 Yenepoya University, Mangalore, India; 11 Center for the Study of the State and Society, Buenos Aires, Argentina; 12 Ministry of Health of Argentina, Buenos Aires, Argentina; 13 Institute for Evidence-Based Healthcare, Bond University, Gold Coast, Australia; 14 Dalla Lana School of Public Health, University of Toronto, Toronto, Canada; 15 Lao-Oxford-Mahosot Hospital-Wellcome Trust Research Unit (LOMWRU), Microbiology Laboratory, Mahosot Hospital, Vientiane, Lao People’s Democratic Republic

## Abstract

Over recent years, the research community has been increasingly using preprint servers to share manuscripts that are not yet peer-reviewed. Even if it enables quick dissemination of research findings, this practice raises several challenges in publication ethics and integrity. In particular, preprints have become an important source of information for stakeholders interested in COVID19 research developments, including traditional media, social media, and policy makers. Despite caveats about their nature, many users can still confuse pre-prints with peer-reviewed manuscripts. If unconfirmed but already widely shared first-draft results later prove wrong or misinterpreted, it can be very difficult to “unlearn” what we thought was true. Complexity further increases if unconfirmed findings have been used to inform guidelines. To help achieve a balance between early access to research findings and its negative consequences, we formulated five recommendations: (a) consensus should be sought on a term clearer than ‘pre-print’, such as ‘Unrefereed manuscript’, “Manuscript awaiting peer review” or ‘’Non-reviewed manuscript”; (b) Caveats about unrefereed manuscripts should be prominent on their first page, and each page should include a red watermark stating ‘Caution—Not Peer Reviewed’; (c) pre-print authors should certify that their manuscript will be submitted to a peer-review journal, and should regularly update the manuscript status; (d) high level consultations should be convened, to formulate clear principles and policies for the publication and dissemination of non-peer reviewed research results; (e) in the longer term, an international initiative to certify servers that comply with good practices could be envisaged.

## Background

Over recent years, the research community has been increasingly using preprint servers to share manuscripts that have not yet been peer-reviewed, enabling quick dissemination of research findings, and in some cases to obtain peer feedback to improve the final version submitted to a journal [1]. Relevant international stakeholders provide guidance on ethics and integrity in scientific publications, in particular the Committee on Publication Ethics (COPE) and the International Committee of Medical Journal Editors (ICMJE). However, issues related to publication ethics and integrity become more complicated, when a form of publication has already occurred before peer review and is available to the public. Furthermore, calls to rapidly share research results to inform public health emergencies including COVID19, have introduced additional layers of complexity [2–4]. For instance, according to the World Health Organization (WHO) Working Group on Ethics and COVID19, researchers generating information with the potential to aid response efforts have an ethical obligation to share it “without waiting for publication in scientific journals”, and as soon as the information “is quality-controlled for release (e.g., peer-reviewed)” [5]. These recommendations have an inherent conflict, as the quality control of the peer-review process is managed by the scientific journals whose publication timelines should not be waited for. The pursuit of rapid dissemination also comes into conflict with the need to comply with adequate publication standards [2].

### Preprint platforms: benefits and risks

Preprints (i.e. preliminary reports of work not yet subject to peer review) are increasingly posted online on websites such as the well-known MedRxiv, arXiv and bioRxiv [6–8], as well as other servers. These can be valuable mechanisms to facilitate rapid communication within the international scientific community, and trigger early, fruitful and transparent discussion among peers, while waiting for the scientific work to undergo a journal’s peer review. A cross-sectional study of preprint policies among the 100 clinical journals with the highest impact factors showed that 86% of journals allow for submitted articles to be previously posted as preprints, making researchers less concerned that posting a manuscript on a preprint server will disqualify it from further publication [1]. However, it remains important to thoroughly understand and accurately describe the nature and purpose of preprints. This is why arXiv explicitly states that material is “not peer-reviewed by arXiv” and” they should not be relied upon without context to guide clinical practice or health-related behavior and should not be reported in news media as established information without consulting multiple experts in the field” [7]; and bioRxiv clarifies that “articles are not peer-reviewed, edited, or typeset before being posted online” [8]. Preprints posted in MedRxiv, arXiv and bioRxiv include a small header or footer on each page with a warning that the article has not undergone peer review [6–8]. MedRxiv also explicitly states that due to the inherent nature of “preliminary reports of work that have not been certified by peer review”, preprints “should not be relied on to guide clinical practice or health-related behavior and should not be reported in news media as established information” [6]. This is an important caveat as there is no assurance that preprints have not had any external quality control when they are made publicly available. However, it is unlikely that it will always be carefully read—for instance, when preprints are circulated via social media.

Though peer review remains the de facto source of quality-assurance in scientific publishing [9], it does not always prevent inaccurate reports from entering the scientific literature [10–12], and mechanisms to ensure reviewer access to original data sets or codes for analysis to strengthen the quality of review are not systematically in place. This has become particularly evident during the pandemic. Smith and colleagues noted in March 2021 that in a rush to disseminate information about COVID19, diverse inaccuracies have been published, causing inappropriate changes in clinical care, ineffective public health responses, and increasing anxiety in communities [13]. It is also likely that the rush to publish COVID19 manuscripts is causing shortages of qualified reviewers. The vast and growing volume of submissions related to COVID19 is precipitating reviewer fatigue. Despite the limitations, peer review can help to mitigate overinterpretation and misreporting of results, and reduce the proportion of poor-quality and inaccurate publications [11–13]. Therefore, when it comes to pre-prints, both authors and readers would benefit from guidance about their appropriate dissemination and interpretation, and on reliable servers for posting and finding preprints.

The WHO has established a platform bringing together approved clinical trial registries [14], and a similar initiative for pre-print servers may be necessary. The Committee on Publication Ethics (COPE) and the International Committee of Medical Journal Editors (ICMJE) could play an essential role in setting high ethical standards for reporting and disseminating research findings—including those that have not yet been peer-reviewed-, intending to promote integrity along all phases of research dissemination.

### Preprints: what’s in a name

Despite the caveats provided in various servers, preprints can still be confused with peer-reviewed manuscripts. The name “pre-print” itself may further encourage misunderstanding. The term implies that the manuscript will be printed or published, thus inferring a level of quality sufficient for publication. It may also raise expectations that the manuscript *should*, or ever *will in any case be* printed. Additionally, “pre-print” sounds very close to “Epub ahead of print", which indicates a peer-reviewed article that is being listed electronically before being typeset [15].

For some users, especially those who are not involved in the scientific publishing process, such as journalists, politicians, policy-makers, and the general public, this language implicitly suggests a sort of *imprimatur*, i.e. that the contents have already be approved, which may increase the probability that they are widely promoted and disseminated before a robust external evaluation. A clearer term should be chosen instead of ‘preprint’, such as ‘Unrefereed manuscript’ or “Manuscript awaiting peer review” or ‘’Non-reviewed manuscript”, to avoid preventable misunderstandings.

### Hunger for scientific information in times of COVID19

The COVID19 pandemic has created a global “hunger” for scientific information to inform policy and the general public. In the 5 months prior to the COVID19 era (1/8/2019 to 31/12/2019), 14,078 preprints were posted in MedRxiv and bioRxiv. This number increased by 61.2% to 22,691 in the first 5-months of the COVID19 era (1/1/2020 to 31/5/2020), including both COVID19 and non-COVID19-related preprints. Preprints have become an important source of information for a variety of stakeholders interested in COVID19 research developments, including traditional media, social media, and policy-makers. But the philosophy of preprints, i.e. that errors will get fixed over time as the scientific community crowdsources and opines on the findings [22], may be unknown to or misunderstood by many of their new users, including politicians, journalists, “influencers”, prescribers, policy-makers—and sometimes even researchers. Nowadays, research appraisal and synthesis often occur before a decision is reached on publication, with the risk of leading to “irresponsible dissemination, as flawed studies are picked up by the media” [2]. For example, suppose clinical trial findings are only available as preprints. In that case, a cautionary approach should be taken to the interpretation of their findings, which should not be used as the sole basis for introducing a new therapeutic or preventive intervention into practice and policy. Lack of caution may have been a factor in the publication and withdrawal of early evidence on hydroxychloroquine efficacy in COVID19 [16–20]. Numerous papers related to COVID19 cite preprint materials, and it is not always explicitly stated that these are not peer-reviewed and should be cautiously and critically interpreted [21]. Additionally, if unconfirmed but widely shared first-draft results later prove to be wrong or misinterpreted, it can be very difficult for people to “unlearn” what they thought was true [22], because of ‘anchoring bias’, which comes into play when individuals prioritize information and data that support their initial impressions, even when first impressions are wrong [23]. Complexity may further increase if such unconfirmed findings had been used to inform guidelines; and if pre-prints remain pre-prints indefinitely and never get formally published, either because they are rejected or not even submitted.

We need a balance between early access to research findings and its unwanted negative consequences, such as the rushed adoption of policies unsupported by evidence on efficacy and safety, and misplaced or unrealistic expectations from the public, policy-makers and stakeholders [16, 24–27]. Therefore, we contend that *all* preprint servers should state, as does MedRxiv [6], that “Preprints are preliminary reports of work that have not been certified by peer review. They should not be relied on to guide clinical practice or health-related behaviour and should not be reported in news media as established information”. The caveat should not be divorced from preprints when they circulate, thus this statement should be prominent on the first page of each preprint, and each page should include a red watermark stating ‘Caution—Not Peer-Reviewed’ [10, 15]. This call is in line with a statement from the Editors of The Lancet, who confirmed the journal policy to make preprints a permanent offering, while applying “a more obvious watermark stating that these are preprints and not peer-reviewed” [28].

### A call for scientific integrity standards

While preprints can help to make new evidence-based knowledge rapidly available to the scientific community, and perhaps improve scientific transparency [15], these benefits can be undermined by harms caused by the release, dissemination, and misuse of unreliable evidence. It is not a foregone conclusion that the potential benefits of preprints always outweigh the risks of harm [10, 29].﻿ It is also crucial that preprints are accompanied by a minimal set of essential, ethics-related information. However, this is not always the case. For instance, only two-thirds of research paper authors who submitted to the *Lancet* Group journals and opted to post pre-prints at submission, had posted all this information, i.e. ethics approval if needed, declaration of interests, funding statement, and prospective registration for randomised controlled trials [28]. Thus, the various stakeholders that currently encourage the use of ‘preprints’, including research institutions and research funding agencies [30], should also encourage improvement of ethical standards, appropriate terminology, warnings regarding interpretation and dissemination, and recognition of limitations. Given the rapid and growing importance and use of preprint servers, particularly concerning the ongoing pandemic, the time is ripe for the scientific community to agree on “Good Preprint Practices” formally. Adequate and harmonized scientific quality and integrity standards [10] should be defined, as they have been for other critical research and reporting activities [31], and appropriate mechanisms for implementation and enforcement of such good practices should be identified and implemented. In particular, we propose the following specific recommendations:

- As the term “preprint” may cause misunderstanding about the nature of these manuscripts, consensus should be urgently sought on a clearer term, such as ‘Unrefereed manuscript’ or “Manuscript awaiting peer review” or ‘’Non-reviewed manuscript”.
- Key caveats about unrefereed manuscripts (i.e., that they are preliminary reports of work that have not been subject to peer review; that they should not be relied on to guide clinical practice or health-related behaviour; and that they should not be reported in news media as established information), should be prominent on the first page of each preprint, and each page should include a red watermark stating ‘Caution—Not Peer Reviewed’.
- Pre-print servers should require authors to certify that the manuscript will imminently be -or is- submitted to a peer review journal; to regularly update the status of the manuscript (e.g. submitted, rejected, re-submitted, published with DOI); and to disclose their pre-print history.
- High level consultations should be convened with relevant stakeholders, including but not limited to the ICMJE, COPE and WHO, to formulate clear principles and policies for the publication and dissemination of non peer-reviewed research results, and to further disseminate such principles and policies to the scientific community.
- In the longer term, an initiative to certify servers that comply with agreed good practices could be envisaged.

## Data Availability

Not applicable.

## Abbreviations

COPE: Committee on Publication Ethics
ICMJE: International Committee of Medical Journal Editors
WHO: World Health Organization
